# Care team and practice-level implementation strategies to optimize pediatric collaborative care: study protocol for a cluster-randomized hybrid type III trial

**DOI:** 10.1186/s13012-022-01195-7

**Published:** 2022-02-22

**Authors:** David J. Kolko, Elizabeth A. McGuier, Renee Turchi, Eileen Thompson, Satish Iyengar, Shawna N. Smith, Kimberly Hoagwood, Celeste Liebrecht, Ian M. Bennett, Byron J. Powell, Kelly Kelleher, Maria Silva, Amy M. Kilbourne

**Affiliations:** 1 Department of Psychiatry, University of Pittsburgh School of Medicine, Pittsburgh, PA USA; 2 Department of Pediatrics, Drexel University College of Medicine and St. Christopher’s Hospital for Children, Philadelphia, PA USA; 3 PA Medical Home Program, PA Chapter, American Academy of Pediatrics, Media, PA USA; 4 Department of Statistics, University of Pittsburgh, Pittsburgh, PA USA; 5 Department of Health Management & Policy, School of Public Health, University of Michigan, Ann Arbor, MI USA; 6 Department of Child and Adolescent Psychiatry, New York University Langone Health, New York, NY USA; 7 Department of Learning Health Sciences, University of Michigan Medical School, Ann Arbor, MI USA; 8 Departments of Family Medicine and Psychiatry and Behavioral Sciences, University of Washington, Seattle, WA USA; 9 Center for Mental Health Services Research, Brown School, Washington University in St. Louis, One Brookings Drive, St. Louis, MO 63130 USA; 10 Division of Infectious Diseases, John T. Milliken Department of Medicine, Washington University School of Medicine, Washington University in St. Louis, St. Louis, MO USA; 11 Department of Pediatrics, College of Medicine, The Ohio State University, Columbus, OH USA; 12 Nationwide Children’s Hospital Research Institute, Columbus, OH USA; 13 Allegheny Family Network, Pittsburgh, PA USA; 14 VA Ann Arbor Healthcare System, Ann Arbor, MI USA

**Keywords:** Collaborative care model, Facilitation, Implementation strategies, Mechanisms, Team, Leadership

## Abstract

**Background:**

Implementation facilitation is an effective strategy to support the implementation of evidence-based practices (EBPs), but our understanding of multilevel strategies and the mechanisms of change within the “black box” of implementation facilitation is limited. This implementation trial seeks to disentangle and evaluate the effects of facilitation strategies that separately target the care team and leadership levels on implementation of a collaborative care model in pediatric primary care. Strategies targeting the provider care team (TEAM) should engage team-level mechanisms, and strategies targeting leaders (LEAD) should engage organizational mechanisms.

**Methods:**

We will conduct a hybrid type 3 effectiveness–implementation trial in a 2 × 2 factorial design to evaluate the main and interactive effects of TEAM and LEAD and test for mediation and moderation of effects. Twenty-four pediatric primary care practices will receive standard REP training to implement Doctor–Office Collaborative Care (DOCC) and then be randomized to (1) Standard REP only, (2) TEAM, (3) LEAD, or (4) TEAM + LEAD. Implementation outcomes are DOCC service delivery and change in practice-level care management competencies. Clinical outcomes are child symptom severity and quality of life.

**Discussion:**

This statewide trial is one of the first to test the unique and synergistic effects of implementation strategies targeting care teams and practice leadership. It will advance our knowledge of effective care team and practice-level implementation strategies and mechanisms of change. Findings will support efforts to improve common child behavioral health conditions by optimizing scale-up and sustainment of CCMs in a pediatric patient-centered medical home.

**Trial registration:**

ClinicalTrials.gov, NCT04946253. Registered June 30, 2021.

**Supplementary Information:**

The online version contains supplementary material available at 10.1186/s13012-022-01195-7.

Contributions to the literature

- This statewide trial is one of the first to test implementation strategies at the team and leadership levels in diverse primary care practices to improve common child behavior health conditions.
- Findings will advance our knowledge about how care team and organizational level strategies work to best implement a collaborative care model (CCM).
- Such evidence will optimize efforts to scale up and sustain CCMs in pediatric patient-centered medical homes.

## Background

### Benefits and challenges of integrated care models to improve pediatric behavioral health

Fewer than half of all children with disruptive behavior disorders (DBD; 46%) or attention deficit hyperactivity disorder (ADHD; 48%) receive treatment [1], so many may exhibit long-term impairments [2]. Proactive intervention by pediatric primary care providers (PCPs) in patient-centered medical homes may prevent or attenuate these impairments [3–8]. Integrated care approaches, such as collaborative care models (CCM), target behavioral health (BH) problems in health care settings [9–12]. Meta-analyses show that these approaches improve clinical outcomes in adults [12–16], especially women and people of color [17, 18] and, to a lesser extent, in children/youth [3, 8].

Based on Wagner’s Chronic Care Model [19], CCMs include core components (e.g., delivery system redesign, self-management support) [11, 20] to support key features that include team-based care, progress monitoring, and brief evidence-based interventions [3, 11, 21]. CCM teams typically include PCPs, care managers/coordinators, and a mental health specialist (e.g., psychiatrist) who provides consultation and decision support for complex cases, with most functions coordinated and delivered by the care manager [11]. Because CCMs are complex multi-component interventions, their implementation presents practical challenges at multiple levels [20, 22–31].

### Multilevel determinants of CCM implementation

This study draws upon the EPIS framework to organize our understanding of barriers and facilitators (i.e., determinants) [32, 33]. Common inner context determinants of CCMs include those related to individual provider characteristics (e.g., attitudes, self-efficacy), leadership, and organizational characteristics [20, 25–30, 34]. Team functioning is also a key determinant in team-based service settings like primary care [5]. Implementing evidence-based practices (EBPs) requires teams to adapt to respond to new demands. Team functioning includes affective, behavioral, and cognitive processes and states (e.g., trust, coordination, shared knowledge) and is associated with implementation and patient outcomes [35–39]. At the leadership and organizational levels, successful CCM implementation requires supportive leadership, positive organizational climate and culture, and strong implementation climate [26]. Organizations that reinforce EBP use and provide ongoing support to providers set the stage for successful adoption [40, 41]. Effective leaders encourage positive views about the innovation, leverage time, and resources to support it and may directly champion implementation [41–48].

Achieving public health impact requires scale-up and sustainment of CCMs, especially in low-resource areas [3, 22–24, 28, 31, 49]. However, the current science about how to target these determinants provides few answers. None of the trials in the aforementioned pediatric meta-analysis tested the effects of specific implementation strategies on provider or patient outcomes or their mechanisms of action [3, 8]. We lack effective implementation strategies to guide the scale-up and maintenance of CCMs in pediatric medical homes [50].

### Implementation facilitation can promote uptake of CCMs

Multi-level implementation strategies targeting CCM determinants can improve implementation outcomes [51, 52]. One promising approach is implementation facilitation, a type of interactive assistance designed to overcome barriers and leverage strengths to foster EBP implementation [52, 53]. Facilitation, based on the PARiHS framework [54], is a discrete and multifaceted implementation strategy intended to be flexible and responsive to local circumstances [55, 56]. It is hypothesized to promote organizational learning [56], although our understanding of the specific mechanisms through which facilitation operates is limited [56–59].

Facilitation has been broadly operationalized in two forms, sometimes described as external and internal facilitation [60]. External facilitation involves the use of a facilitator outside of the organization who provides ongoing consultation, coaching, and support to enhance the clinical competencies of providers [52, 60–64]. Facilitation strategies that support front-line providers’ capacity to adopt and deliver a CCM have improved uptake, fidelity, and clinical outcomes in mental health and primary care settings [53, 61, 62, 65, 66].

Internal facilitation involves supporting and training leaders to serve as facilitators within their settings who can bolster EBP delivery by reducing organizational barriers [52, 53, 60, 67, 68]. These strategies (e.g., mentoring managers to adapt workflows and support/reinforce EBP delivery) are designed to reduce organizational barriers and leverage resources to support EBP integration. Internal facilitation has augmented the impact of external facilitation on uptake in community settings, but not always [51, 53, 65, 69]. Generally, research has shown benefits of internal facilitation on EBP competencies/fidelity with providers in adult primary care [66, 70] and mental health agencies [58, 71, 72].

Some studies, however, have not found incremental benefits for external or internal facilitation, and others have found more limited benefits of internal facilitation in typical, low-resource community practices [53, 65, 69]. Internal facilitation has primarily been examined in combination with external facilitation, so its separate effects are relatively unknown. Importantly, most studies have used facilitation to target multiple levels (e.g., individual, team, leadership, organization) simultaneously, including using “blended” or “two-tiered” facilitation models [73, 74], limiting our understanding of mechanisms of change within the “black box” of implementation facilitation [57–59].

Care team providers and practice leaders have different levers of influence that can aid in sustainment of EBPs [22, 24]. At this point, no implementation trial to our knowledge has evaluated the separate and combined effects of facilitation strategies targeting the care team and facilitation strategies targeting practice leadership. It is plausible that these two facilitation strategies have synergistic effects on implementation outcomes by potentiating greater engagement of their respective target mechanisms [75, 76]. Strategies targeting the provider care team should engage team-level mechanisms (e.g., team functioning) [35], whereas strategies targeting leaders should engage organizational mechanisms (e.g., implementation climate, implementation leadership) [77, 78]. Testing mechanisms of action of implementation strategies at specific levels will advance implementation science [79–84].

## Current study

We propose to disentangle and further refine facilitation strategies targeting the care team and leadership levels to support implementation of a CCM in pediatric primary care. We will conduct a cluster-randomized, hybrid type 3 effectiveness–implementation trial [85] using a 2 × 2 factorial design to test the main and interactive effects of implementation strategies that target the care team level (TEAM) or leadership level (LEAD) on implementation and clinical outcomes. All practices will first receive standard implementation strategies based on the Replicating Effective Programs (REP) model [86]. Practices then will be randomized to four conditions: (1) Standard REP only; (2) TEAM, (3) LEAD, and (4) TEAM + LEAD.

Standard REP is a low-cost and low-burden strategy consisting of a tailored intervention manual, didactic training, and brief technical support [87–89]. REP is based on the Centers for Disease Control and Prevention’s Research-to-Practice Framework [86, 90, 91] and derived from Social Learning Theory [92] and Rogers’ diffusion model [93]. It is easily scalable in most community-based practices. Although standard REP only may help some sites to adequately adopt an EBP, evidence suggests it is unlikely to be sufficient in many lower-resourced settings, and augmentations to REP may be necessary [53]. In this study, we will evaluate the effects of augmenting REP with two different types of facilitation (TEAM and LEAD) targeting different levels, audiences, and mechanisms.

TEAM facilitation is informed by existing approaches to facilitation, including external facilitation [52, 53, 67], practice facilitation [62, 64, 94, 95], and coaching [63, 64], in which an outside expert helps providers improve EBP uptake. TEAM also incorporates strategies from team development interventions (i.e., team building [96, 97], team training [98, 99], debriefing [100, 101]) to improve care team functioning and effectiveness. TEAM aims to improve implementation outcomes by targeting provider clinical competencies, team functioning, and team integration/quality.

LEAD is based on the Kirchner [67] and Kilbourne et al. [102] internal facilitation role. It focuses on reducing organizational barriers to implementation by promoting practice champions who sustain the EBP. LEAD aims to improve implementation outcomes by targeting implementation climate [77, 103, 104] and implementation leadership [105, 106].

### The EBP: DOCC

Doctor–Office Collaborative Care (DOCC) is a cross-diagnosis intervention for treating DBDs and comorbid ADHD in community pediatric practices [107–109]. DOCC is based on the CCM’s core components adapted for the medical home [11, 12, 50, 110]. In randomized clinical trials, DOCC improved service access (99% vs. 46%) and completion (77% vs. 12%), personalized behavioral and ADHD targets, externalizing and ADHD symptoms, remission rates, family satisfaction, and provider self-efficacy and effectiveness, with most symptom resolution in fewer than 11 contacts [107–109, 111]. We also documented lower BH care costs at 12-month follow-up for DOCC [112].

In this trial, the DOCC package includes an implementation guide and a provider treatment manual. The implementation guide includes resources and guidelines for key care management processes that support the six CCM principles: organizational support, delivery system redesign (e.g., team roles, workflows), clinical decision support (e.g., use of standardized rating scales), clinical information systems (e.g., patient registry), self-management support (e.g., workbook), and community resources. The treatment manual includes DBD modules with skills for caregivers and children (e.g., anger management, parenting) and ADHD modules that address psychoeducation, shared decision-making, and medication recommendations. The care team is responsible for assessing treatment progress, individualizing session frequency and treatment dose, and coordinating with other services.

### Study aims

This trial seeks to accelerate understanding of the implementation strategies needed to deliver and scale up CCMs in pediatric primary care. The specific aims are the following:

#### Aim 1: test the effects of TEAM and LEAD on implementation outcomes and child clinical outcomes

Our implementation outcomes are DOCC service delivery, specifically the number of encounters for each case (primary), and change in practice-level care management competencies (exploratory) at 4 timepoints (6, 12, 18, and 24 months). We will also examine secondary clinical outcomes (change in severity of child symptoms). We hypothesize that augmenting REP with TEAM alone or LEAD alone is superior to Standard REP, and that TEAM + LEAD is superior to all other conditions because it targets both levels.

#### Aim 2: test for target engagement in each implementation condition and if hypothesized mechanisms mediate the effects of TEAM and LEAD on implementation outcomes

Our hypothesized mechanisms for TEAM are team functioning and effectiveness; hypothesized mechanisms for LEAD are implementation leadership and implementation climate. We hypothesize that each condition will have main effects on one or both of its targets. We also expect that the effects of TEAM and LEAD on outcomes will be mediated by their hypothesized targets.

#### Aim 3: examine provider-, practice-, and family-level moderators of the effects of TEAM or LEAD

Proposed provider-level moderators of TEAM effects are attitudes about delivering BH care and care manager discipline. Proposed practice-level moderators of LEAD are baseline implementation leadership and climate. Lastly, proposed family-level moderators of TEAM or LEAD are caregiver gender, caregiver race/ethnicity, and child baseline symptom severity.

## Methods

### Study design

**Fig. 1:**
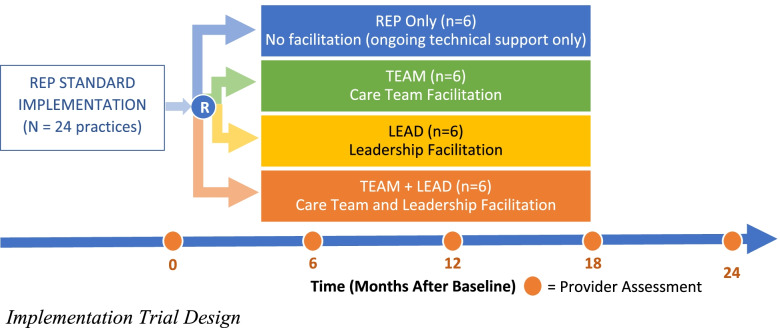
Implementation trial design

We propose a hybrid type 3, cluster-randomized effectiveness–implementation trial in a 2 × 2 factorial design in 24 pediatric primary care practices across Pennsylvania. After all sites receive Standard REP, they will be randomized to one of four conditions: (1) Standard REP only (continued technical assistance), (2) TEAM, (3) LEAD, or (4) TEAM + LEAD. Figure 1 outlines the trial design. Care teams will deliver DOCC to 25 children with elevated behavioral problems and their caregivers. We will collect longitudinal data from practice staff and caregivers. SPIRIT, CONSORT, and TIDieR checklists [113–115] are in Supplemental File 1, and CONSORT flow diagrams are in Supplemental File 2. All procedures were approved by the University of Pittsburgh Institutional Review Board.

### Setting and participants

#### Practices

Study sites will be 24 primary care practices affiliated with the Pennsylvania Chapter of the American Academy of Pediatrics Medical Home Program (PA-MHP) [4, 7, 116]. These practices are heterogenous with respect to their size, location, health system, insurance mix, and population diversity. PA-MHP leadership identified eligible and interested practices and organized orientation meetings with key practice leaders.

#### Professionals

##### Practice leaders

In each practice, we will enroll the lead PCP or medical director (*N* = 24) and practice manager (*N* = 24). Individuals in these positions are responsible for decision-making and management tasks at their sites. Lead PCPs and practice managers in practices randomized to LEAD will participate in Leadership Facilitation.

##### Primary care providers (PCPs)

We seek to enroll all eligible PCPs at participating practices (*M* = 4.4 PCPs per practice, range: 1–10) to maximize the likelihood that families have an enrolled PCP and enhance generalizability. All PCPs will be provided access to training and encouraged to deliver DOCC. PCPs in practices randomized to TEAM will participate in Team Facilitation.

##### Care managers (CMs)

In each practice, we will enroll the individual acting as the practice’s behavioral health resource to serve as the CM (*N* = 24). Individuals in this role may vary in professional discipline (e.g., nursing, mental health) and experience delivering psychosocial interventions. The CM will deliver DOCC in collaboration with PCPs and the care team. CMs in practices randomized to TEAM will participate in Team Facilitation.

#### Caregivers

We will enroll up to 25 caregivers of 5–12-year-old children in each practice for a possible total of 600 caregivers. Eligibility criteria are (1) child age (5–12 years), (2) parent/guardian with parental rights, and (3) child meets clinical cutoff on 7-item externalizing problems scale of the Pediatric Symptom Checklist-17 (PSC-17) [117]. Based on prior trials, we expect to recruit more female than male caregivers and for participants to vary in race/ethnicity by practice. Our primary informant is the caregiver due to the children’s young age and ethical concerns about assessing children without face-to-face contact.

### Implementation conditions and strategies

The four implementation conditions are shown in Fig. 1. Table 1 lists the mechanisms of change at the individual, team, and practice levels targeted by each condition. Supplemental File 3 shows the ERIC implementation strategies included within each condition and their hypothesized mechanisms of action (Supplemental Table 1) and the specific actions within each condition (Supplemental Table 2).

**Table 1:** Implementation conditions and targets by level

Level	Implementation condition	Target/mechanism
Individual provider	Standard REP	Self-efficacy
		Attitudes
Care team	TEAM facilitation	DOCC skill
		Fidelity
		Affective, behavioral, and cognitive functioning
		Team effectiveness
Practice	LEAD facilitation	Implementation climate
		Implementation leadership

#### Standard REP (no facilitation)

All practices will receive DOCC manuals and training and participate in the same study initiation meetings (e.g., staff introductions, orientation to study procedures). Each provider will receive access to DOCC virtual training, which includes content and care processes organized into nine clinical topics, each with brief videos and post-training knowledge quizzes. Providers can use the platform to contact study staff for technical assistance and clarification or discussion of training content. The training platform will record data on progress, completion, and performance (e.g., modules accessed, quiz scores), and providers will receive continuing education credits. All sites will receive ongoing technical assistance during the implementation phase.

#### Care team facilitation (TEAM)

TEAM is a phased approach designed to improve providers’ skill in using DOCC, teamwork quality, and team effectiveness. The TEAM facilitator is a licensed clinician who delivered DOCC in a prior trial and has lived experience as a consumer of integrated care for a child with ADHD. TEAM facilitation will occur via a graded schedule of videoconference calls over 18 months (weekly to bimonthly).

In the first phase of TEAM, the facilitator will engage the care team in identifying barriers and facilitators, enhancing motivation to use DOCC, and setting goals for implementation in their practice. The second phase focuses on reviewing and revising roles, responsibilities, and workflows within the team to improve collaboration, coordination, and use of DOCC. As part of this phase, the facilitator will support the team in developing effective communication and problem-solving skills. The third phase focuses on increasing the team’s competency and fidelity to DOCC through ongoing training in treatment content (e.g., didactics, modeling, role plays), structured reviews of patient progress, audit and feedback on use of the patient registry, consultation on challenging cases, and support in overcoming barriers and balancing model adaptations and fidelity. The last phase of TEAM focuses on the team’s capacity to sustain and continually improve DOCC in their practice. The facilitator will conduct structured team debriefings and encourage the team to identify and address potential problems in team processes. The facilitator will also guide the team in reflecting on implementation, reviewing and revising implementation goals, and planning for sustainability and continuous quality improvement.

#### Practice leadership facilitation (LEAD)

LEAD is a phased approach designed to strengthen practice leadership’s capacity to lead change and overcome practice-level barriers. The LEAD facilitator and consulting psychiatrist is a pediatrician and faculty member with expertise in pediatric integrated care, consulting with PCPs and CMs, ADHD medication management, and behavior problems. LEAD will follow the same graded schedule of videoconference calls as TEAM.

In the first phase of LEAD, the facilitator will engage leadership in identifying barriers and facilitators to DOCC, learning about the model, and setting goals for practice implementation. The second phase focuses on aligning implementation with practice priorities, reinforcing leaders’ attention to DOCC, and creating an action plan to reach implementation goals. During the third phase, the facilitator will engage leadership in reducing barriers by leveraging relationships, aligning fiscal resources with DOCC core activities, engaging community partners, and sharing lessons learned from other practice leaders. The last phase focuses on leadership’s capacity to sustain and continually improve DOCC in their practice by encouraging them to think strategically about system-level barriers and facilitators and plan for sustainability and ongoing monitoring of progress. The facilitator will guide leaders to reflect on implementation goals and work to transfer responsibility for DOCC use in their practice.

#### Team and leadership facilitation (TEAM + LEAD)

TEAM + LEAD combines both approaches described above to improve distinct but potentially complementary targets at the team and practice levels. Practices randomized to TEAM + LEAD will participate in all the above activities, and the TEAM and LEAD facilitators will work together to align the goals and actions of the care team and leadership.

### Procedures

#### Randomization

For feasibility, the trial will occur in three cohorts. Randomization will occur at the beginning of each planned cohort. If possible, we will stratify the practices in each cohort by Medicaid rate before randomizing. Randomization will be conducted by the project’s data manager using random number generation in SAS. Practices will be informed of randomization after baseline data collection is completed. Research staff who have contact with participants will be unaware of implementation condition.

#### Caregiver recruitment and screening

Caregivers of children visiting the practice will be informed about the study using multiple methods (e.g., posters, brochures, PCP referral). Recruitment materials, available in English and Spanish, direct caregivers to their PCP and a website with more information and a short orientation video. Caregivers complete an online screening process. Eligible caregivers will then be given login credentials to access and complete an online consent and the baseline assessment via a smartphone, tablet, or computer. Caregivers who cannot read or write and caregivers without a smartphone or computer are directed to call the study coordinator to complete the screening and consent process.

#### Professional and caregiver assessments

Practice staff will complete assessments at 0, 6, 12, 18, and 24 months. Caregivers will complete assessments at 0, 3, 6, and 12 months and provide consent for their child’s teacher to complete rating scales at each timepoint. Measures are listed in Table 2. All assessments can be completed online, on paper, or by phone with research staff. Participants will be paid for each completed assessment. We estimate high retention rates for practice staff (> 90%) and caregivers (> 85%) based on our previous trials (91–92%) [107–109]. We will use strategies from prior trials (e.g., trained staff, on-time bonus payments) to support retention.

**Table 2:** Primary assessment constructs, measures, sources, and timepoints by study aim

Construct	Measure	Source	Month					
			0	3	6	12	18	24
**Aim 1: implementation outcomes**								
Total # of DOCC sessions received	SPL	Provider notes	During services					
Care management competencies	MHPRI	All staff	x		x	x	x	x
(Acceptability, appropriateness, feasibility)	(AIM, IAM, FIM)	All staff	x		x	x	x	x
**Aim 1: clinical outcomes**								
Child symptom severity	VADPRS	Caregiver	x	x	x	x		
Child symptom severity	VADTRS	Teacher	x	x	x	x		
Child health-related quality of life	PEDS-QL	Caregiver	x	x	x	x		
**Aim 2: mediators**								
Care team functioning	PCTDS (TDM)	All staff	x		x	x	x	x
Care team integration/quality	PICS	Caregiver	x	x	x	x		
Implementation climate	ICS (ISM)	All staff	x		x	x	x	x
Implementation leadership	ILS (ISM)	All staff	x		x	x	x	x
**Aim 3: moderators and other variables**								
Practice characteristics (e.g., size, % Medicaid)	PINS	Manager	x					
Staff characteristics (e.g., training, education)	SIF	CM	x					
Child characteristics (e.g., gender, minority status)	FIF	Caregiver	x					
Attitudes about delivering behavioral health care	PBS	All staff	x		x	x	x	x

Secondary constructs/measures are indicated by parentheses

*SPL* Services Provided Log, *MHPRI* Mental Health Practice Readiness Inventory, *AIM* Acceptability of Intervention Measure, *IAM* Intervention Appropriateness Measure, *FIM* Feasibility of Intervention Measure, *VADPRS* Vanderbilt ADHD Diagnostic Parent Rating Scale, *VADTRS* Vanderbilt ADHD Diagnostic Teacher Rating Scale, *PEDS-QL* Pediatric Quality of Life, *PCTDS* Primary Care Team Dynamics Survey, *TDM* Team Development Measure, *PICS* Pediatric Integrated Care Survey, *ICS* Implementation Climate Scale, *ISM* Inner Setting Measures, *ILS* Implementation Leadership Scale, *PINS* Practice Information and Needs Survey, *SIF* Staff Information Form, *FIF* Family Information Form, *PBS* Physician Belief Scale

### Measures

#### Background information

At baseline, each practice manager will complete a Practice Information and Needs Survey (PINS) form, and professionals will complete a Staff Information Form (SIF). Caregivers will complete a Family Information Form (FIF) to provide background information, including any other treatment services [118].

#### Implementation outcomes

##### Services Provided Log (SPL)

After each service encounter, providers will record the type of contact (e.g., treatment session, collaborative care meeting), individuals present, intervention content, and plans for next contact [107, 108]. Our primary outcome is the number of DOCC service encounters delivered to each patient by all providers.

##### Mental Health Practice Readiness Inventory (MHPRI)

The MHPRI [119] will document practice-level care management competencies shown to predict care uptake [120, 121].

Practice staff will rate each of the 32 items at the practice-level (0 = no function exists; 1 = some function; 2 = function is complete). We will aggregate all informants’ scores to create a total score for practice achievement of care management competencies.

##### Acceptability of Intervention Measure (AIM), Intervention Appropriateness Measure (IAM), and Feasibility of Intervention Measure (FIM)

Secondary implementation outcomes are DOCC acceptability, feasibility, and appropriateness. They will be assessed with three 4-item scales that have excellent internal consistency and good content validity [122].

#### Clinical outcomes

##### Vanderbilt ADHD Diagnostic Rating Scale

Rating scales will be completed by caregivers (VADPRS) and teachers (VADTRS) at each timepoint [123, 124]. Both versions include symptom severity scales as well as performance/impairment items and have excellent psychometrics.

##### Pediatric Quality of Life (PEDS-QL)

Caregivers will complete the PEDS-QL to measure health-related quality of life [125, 126]. It has excellent reliability and treatment validity and is sensitive to DOCC [107].

#### TEAM and LEAD targets and mediators of implementation outcomes

##### Primary Care Team Dynamics Survey (PCTDS)

Staff will complete the 29-item PCTDS to assess affective, behavioral, and cognitive dimensions of team functioning and overall team effectiveness [127]. It has high reliability and discriminant validity [127].

##### Pediatric Integrated Care Survey (PICS)

Caregiver perceptions of team effectiveness will be assessed with the 6-item PICS [128]. The PICS has good reliability, construct validity, and discriminant validity [128].

##### Team Development Measure (TDM)

As a secondary measure of team functioning, staff will complete the TDM, which assesses specific dimensions of team functioning and provides an overall team development score [129]. The TDM has excellent internal consistency and a clear factor structure [129].

##### Implementation Leadership Scale (ILS)

Staff will complete the ILS to capture the extent to which practice leadership is proactive, knowledgeable, supportive, and perseverant toward DOCC implementation [106]. The ILS has strong psychometric properties and contributes to EBP adoption [105, 106, 130–133]. Minor word changes were made to focus the scale on implementation of EPBs for behavioral health in primary care practices.

##### Implementation Climate Scale (ICS)

Staff will complete the ICS to assess extent to which the practice prioritizes and values implementation of evidence-based practices for behavioral health [104]. The ICS has high reliability and construct validity with organizational measures and is associated with EBP use [77, 104]. As with the ILS, minor word changes were made.

##### Inner Setting Measures (ISM)

As secondary measures, staff will complete three scales assessing overall culture, implementation climate for DOCC specifically, and leadership engagement. These scales have good factor structure, internal consistency, and discriminant validity [134].

#### Potential moderators of the effects of TEAM or LEAD on implementation outcomes

##### Team/provider level

We will test whether negative staff attitudes about BH services (Physician Belief Scale (PBS)) [135] or the CM’s discipline (nursing vs. mental health; SIF) at baseline moderates the effects of TEAM.

##### Practice level

We will examine baseline implementation leadership (ILS) and implementation climate (ICS) as moderators of the LEAD condition.

##### Family level

We will test three family characteristics as moderators of TEAM or LEAD (i.e., caregiver gender, caregiver race/ethnicity, child baseline ADHD severity).

#### Fidelity

##### Fidelity to implementation condition

We will follow Proctor et al.’s recommendations for specifying and reporting implementation strategies [136] and develop implementation manuals for both TEAM and LEAD describing key steps and activities (see Supplemental File 3). Facilitators will track attendance, participation, and activities completed during facilitation calls. They will also record specific barriers, solutions, and next steps.

##### Fidelity to DOCC

Fidelity to DOCC will be documented in two ways. First, we will evaluate dosage for all cases. We define adequate dosage as at least 6 DOCC encounters and at least 1 care management meeting for each case [137, 138]. Second, we will assess fidelity by reviewing audio recordings of DOCC treatment sessions. CMs will upload two session audio recordings for cases that consent to recording via a secure, HIPAA-compliant audio portal. A trained research assistant (unaware of implementation condition) will rate recordings using the Treatment Integrity Rating Form [139].

### Power and sample size

Our power calculations for Aim 1 are based on comparisons of provider and patient outcomes among the six practices in each of the four conditions in the factorial design. For our primary implementation outcome (number of DOCC sessions), we estimate 80% power to detect an effect size (ES) of 0.42. Given our modest practice sample size, we will explore group differences in practice-level CCM core competencies. In our prior trial, large ESs were found for provider changes in behavior management practices (*ES* = 0.78) and perceived competencies (*ES* = 0.77) [107]. For patient outcomes, we assume an ICC of 0.01 in cases treated by the same provider and 20% attrition based on prior work [107, 109]. In a simple RCT with 262 cases/condition, we can detect an effect size as small as 0.25. The effect size for clinical improvement in individualized targets in our prior trial was 0.60, indicating that our sample size provides adequate power to detect group differences in patient outcomes [107].

Power calculations for mediator analyses (Aim 2) are based on simulation studies [140]. Because the sample sizes per cell are modest (6 sites per condition), we are powered to detect large effects. This seems justifiable insofar as smaller effect sizes (e.g., 0.35–0.50) are of less interest given the higher cost of using more intensive combined implementation strategies. Thus, our mediational tests are powered only for a large effect size. For our moderator analyses (Aim 3), we have power 0.80 to detect small effects of *f-square* = 0.03.

### Data management and monitoring

Our IRB-approved protocol specifies plans for data entry, coding, security, and storage of data on a secure server. Our web-based assessment system includes many mechanisms to protect data integrity and promote data quality (e.g., only allowing valid values, warnings of missing responses), and the data manager will maintain detailed data management procedures (e.g., range checks, data quality reports). The Principal Investigator will meet weekly with study personnel to discuss study goals, participant recruitment/retention, progress in data collection and analysis, and any adverse events or participant complaints.

The study team has established procedures for monitoring and managing risks to participants. A Data and Safety Monitoring Board of five external professionals with varied clinical and research expertise will review study reports and summaries of human subjects’ issues annually and submit recommendations regarding study continuation or proposed modifications. Study modifications will be approved by the IRB, and any significant changes in methods will be reported to the project’s program officer and described in an update to the registered protocol on https://ClinicalTrials.gov. The Principal Investigator and approved study team members will have access to the final trial datasets. Study co-investigators and consultants can access the datasets by request after obtaining IRB approval. Per NIMH policy, a deidentified dataset will be prepared for the National Data Archive.

### Statistical analyses

#### Aim 1

Our primary analytical tool for Aim 1 will be a two-way analysis of variance with interaction using mixed-effects linear models for continuous outcomes and generalized linear mixed models for categorical variables. We will use an intent-to-treat approach. We will examine patterns of missingness and, if necessary, use imputation methods using available covariates [141, 142]. For exploratory analyses, we will use variable selection methods such as Lasso and elastic net to get parsimonious models that fit well. Analyses will be conducted at the end of the trial; no interim analyses are planned.

For implementation outcomes, we hypothesize that relative to REP only, TEAM, LEAD, and their interaction will significantly improve (1) the number of DOCC service encounters per case and (2) collaborative care competencies within the practice. We will test effects on outcomes at months 6, 12, 18, and 24. Fixed effects include time, condition, and practice, and random effects will account for nesting. We will conduct planned contrasts to test differences between conditions.

For patient outcomes, we hypothesize that relative to REP only, TEAM, LEAD, and their interaction will significantly improve (1) child symptom severity and (2) quality of life. Fixed effects include time and condition, and random effects will account for nesting. As exploratory analyses, we will run larger models adjusting for demographic and baseline clinical variables and assess goodness of fit.

#### Aim 2

We hypothesize that TEAM practices will show significant gains in our proposed TEAM targets (team functioning, integration/quality) and that LEAD practices will show significant gains in our proposed LEAD targets (implementation leadership, implementation climate). We will use larger models to adjust for demographic and baseline clinical variables, test goodness of fit, and compare different time periods to check model fit. We will test if the association between each condition (TEAM, LEAD) and each implementation outcome is mediated by their respective targets and explore serial mediation.

#### Aim 3

We will test moderation of TEAM effects by provider characteristics, moderation of LEAD effects by practice characteristics, and moderation of TEAM and/or LEAD effects on child outcomes by family characteristics. We will adapt standard tests for interaction for a detailed study of the candidate moderators. We will use Wallace et al.’s method to derive a single optimal linear combination of moderators [143]. The composite moderator typically has a larger *ES* than any one variable; the relative weights of the composite index can be interpreted to determine the relative importance of moderators for practical applications.

### Dissemination plans

Study results will be shared with participating practices, disseminated through scientific conferences and journals, and reported on https://ClinicalTrials.gov. Results will be shared regardless of the magnitude or direction of effects. Authorship decisions will be based on the International Committee of Medical Journal Editors criteria [144].

## Discussion

Effective behavioral health interventions based on the CCM are among the most complex healthcare services provided in primary care. They require reciprocal interactions among primary care staff and specialists, monitoring of symptoms and interventions, shared language and goals, and multilevel infrastructure supports. Implementation facilitation may be necessary to support such interventions even in the best primary care practices. This statewide trial is one of the first studies to test multilevel implementation strategies to improve implementation of a CCM in pediatric primary care. Our factorial design will allow us to test the separate and interactive effects of facilitation strategies targeting the care team and strategies targeting practice leadership on hypothesized team-level and organizational mechanisms of action, furthering our understanding of change mechanisms in implementation [79–84].

This trial is designed to yield the best possible outcomes for community-based primary care practices. It will take place in motivated sites interested in behavioral health interventions who will be engaged by experienced practice network leadership and research teams to deliver this complex intervention. Study parameters were designed in collaboration with practice network leadership and an experienced investigator team to minimize burden and enhance generalizability. We recognize the many challenges to conducting a large community-based trial and the uncertainty underlying the decisions made to address them. We will recruit practices across the state who vary in size, geography, population served, and availability of BH services. Still, it is not clear if practices with fewer resources will participate or if practices will be able to deliver care to children most in need of services.

We considered alternative study designs that might inform large-scale implementation of DOCC (e.g., SMART design, group additive, hybrid type 2) before choosing a factorial design that allows testing two distinct strategies with a feasible sample size. We incorporate implementation science guidelines [55, 136, 145] and prior trial methods (e.g., [53, 63, 65, 66, 70]) to enhance rigor, operationalize our TEAM and LEAD facilitation strategies to enhance reproducibility, and endeavor to advance implementation science by including a sustainability period and planning multilevel tests of mediation [84, 146]. Our study design and parameters may push the limits of our knowledge, but hopefully strike a balance between rigor and feasibility in our efforts to improve implementation of complex BH interventions. Effective strategies for implementing CCMs can enable their scale-up in pediatric primary care and improve children’s behavioral health outcomes.

## Supplementary Information

**Additional file 1:** SPIRIT 2013 Checklist, CONSORT 2010 Checklist, and TIDierR Checklist.

**Additional file 2: Supplemental Figure 1.** CONSORT Flow Diagram for Primary Care Provider Participants. **Supplemental Figure 2.** CONSORT Flow Diagram for Caregiver Participants.

**Additional file 3: Supplemental Table 1.** Implementation Strategies and Targets by Condition. **Supplemental Table 2.** Actions for Implementation Strategies Organized by Phase and Condition.

## Data Availability

Not applicable. No data have been collected yet.
