# Supplementary material for: Care team and practice-level implementation strategies to optimize pediatric collaborative care: study protocol for a cluster-randomized hybrid type III trial
Source: Implement Sci. 2022 Feb 22;17:20. doi: 10.1186/s13012-022-01195-7 (PMC8862323; doi:10.1186/s13012-022-01195-7)
Supplement: Supplementary file 2 — Additional file 2: Supplemental Figure 1. CONSORT Flow Diagram for Primary Care Provider Participants. Supplemental Figure 2. CONSORT Flow Diagram for Caregiver Participants. [file 13012_2022_1195_MOESM2_ESM.docx]

Supplemental File 2

Supplemental Figure 1: CONSORT Flow Diagram for Primary Care Provider Participants

Supplemental Figure 2: CONSORT Flow Diagram for Caregiver Participants

**Figure 1**

*CONSORT Flow Diagram for Primary Care Provider Participants*

Pediatric practice enrollment

(n = 24 practices)

REP Only

(6 practices; n = )

TEAM+LEAD

(6 practices; n = )

Excluded (n = )

Not meeting inclusion criteria (n = )

Declined to participate (n = )

Participants assessed for eligibility

(n = )

Randomized

(n = )

TEAM

(6 practices; n = )

LEAD

(6 practices; n = )

Baseline assessment

(n = )

6m assessment (n = )

Lost to follow-up (n = )

12m assessment (n = )

Lost to follow-up (n = )

24m assessment (n = )

Lost to follow-up (n = )

18m assessment (n = )

Lost to follow-up (n = )

Analyzed (n = )

Excluded from analysis (n = )

6m assessment (n = )

Lost to follow-up (n = )

12m assessment (n = )

Lost to follow-up (n = )

24m assessment (n = )

Lost to follow-up (n = )

18m assessment (n = )

Lost to follow-up (n = )

Analyzed (n = )

Excluded from analysis (n = )

6m assessment (n = )

Lost to follow-up (n = )

12m assessment (n = )

Lost to follow-up (n = )

24m assessment (n = )

Lost to follow-up (n = )

18m assessment (n = )

Lost to follow-up (n = )

Analyzed (n = )

Excluded from analysis (n = )

6m assessment (n = )

Lost to follow-up (n = )

12m assessment (n = )

Lost to follow-up (n = )

24m assessment (n = )

Lost to follow-up (n = )

18m assessment (n = )

Lost to follow-up (n = )

Analyzed (n = )

Excluded from analysis (n = )

**Figure 2**

*CONSORT Flow Diagram for Caregiver Participants*

Excluded (n = )

Not meeting inclusion criteria (n = )

Declined to participate (n = )

Participants assessed for eligibility

(n = )

Baseline assessment

(n = )

3m assessment (n = )

Lost to follow-up (n = )

6m assessment (n = )

Lost to follow-up (n = )

12m assessment (n = )

Lost to follow-up (n = )

Analyzed (n = )

Excluded from analysis (n = )
