# Supplementary material for: Care team and practice-level implementation strategies to optimize pediatric collaborative care: study protocol for a cluster-randomized hybrid type III trial
Source: Implement Sci. 2022 Feb 22;17:20. doi: 10.1186/s13012-022-01195-7 (PMC8862323; doi:10.1186/s13012-022-01195-7)
Supplement: Supplementary file 3 — Additional file 3: Supplemental Table 1. Implementation Strategies and Targets by Condition. Supplemental Table 2. Actions for Implementation Strategies Organized by Phase and Condition. [file 13012_2022_1195_MOESM3_ESM.docx]

Supplemental File 3

Supplemental Table 1: Implementation Strategies and Targets by Condition

Supplemental Table 2: Actions for Implementation Strategies Organized by Phase and Condition

**Table 1: Implementation Strategies and Targets by Condition**

|  | **ERIC Implementation Strategies** | **REP** | **TEAM** | **LEAD** | **TEAM + LEAD** | **Level & Target/Mechanism** |
| --- | --- | --- | --- | --- | --- | --- |
| 1 | Develop educational materials | X | X | X | X | Individual:  DOCC knowledge  Self-efficacy  Attitudes |
| 2 | Develop & organize quality monitoring systems | X | X | X | X |  |
| 3 | Conduct educational meetings | X | X | X | X |  |
| 4 | Distribute educational materials | X | X | X | X |  |
| 5 | Revise professional roles | X | X | X | X |  |
| 6 | Centralize technical assistance | X | X | X | X |  |
| 7 | Implementation facilitation |  | X | X | X | Practice:  Adoption/sustainment (DOCC encounters, care competencies) |
| 8 | Assess for readiness/identify barriers & facilitators |  | X | X | X |  |
| 9 | Assess and redesign workflow |  | X | X | X |  |
| 10 | Promote adaptability |  | X | X | X |  |
| 11 | Purposely reexamine the implementation |  | X | X | X |  |
| 12 | Promote network weaving |  | X |  | X | Care Team:  Affective, behavioral, & cognitive functioning  Effectiveness |
| 13 | Organize clinician implementation team meetings |  | X |  | X |  |
| 14 | Conduct ongoing training |  | X |  | X | Individual:  DOCC skill  Fidelity |
| 15 | Provide ongoing consultation |  | X |  | X |  |
| 16 | Audit and feedback |  | X |  | X |  |
| 17 | Recruit, designate, and train for leadership |  |  | X | X | Practice:  Implementation climate  Implementation leadership |
| 18 | Identify and prepare champions |  |  | X | X |  |
| 19 | Make billing easier |  |  | X | X |  |
| 20 | Engage community resources |  |  | X | X |  |

**Table 2: Actions for Implementation Strategies Organized by Phase and Condition**

| **REP Strategies: All Conditions** | |
| --- | --- |
| 1. Create project website with assessment portals, announcements, web training, and study updates (1, 4) | |
| 1. Meet with practice leadership to introduce project (3) | |
| 1. Create online training curriculum and platform with knowledge quizzes and performance feedback (1) 2. Create manuals for providers and caregivers (1) | |
| 1. Develop patient registry infrastructure (2) | |
| 1. Provide written materials describing team members’ roles, responsibilities, and functions (4, 5) | |
| 1. Provide written materials describing patient registry infrastructure and functions (2, 4) 2. Train staff in CCM principles and DOCC workflows (3, 5) 3. Train staff in DOCC roles and responsibilities (3, 5) 4. Train staff in research participation requirements (3) | |
| 1. Train staff in using patient registry infrastructure and functions (2, 3) 2. Provide physical and online intervention manuals (4) 3. Train staff in DOCC clinical content (3) 4. Conduct weekly calls to staff during training to solicit questions and address concerns (6) 5. Provide monthly open call-in consultation for general questions (6) | |
| 1. Provide on-demand support and technical assistance to providers (6) | |
| 1. Distribute monthly study newsletter with tips and updates (4) | |
|  | |
| **Team Facilitation (TEAM)** | **Leadership Facilitation (LEAD)** |
| *Phase 1: Initiation and Goal-Setting*   1. Schedule meetings (7, 13) 2. Create ground rules (13) 3. Build supportive relationships with team (7) 4. Assess/discuss provider and team-level barriers and facilitators (8) 5. Lead team goal-setting exercise (12) | *Phase 1: Initiation and Goal-Setting*   1. Schedule meetings (7) 2. Build supportive relationships with leaders (7) 3. Teach basics about DOCC (18) 4. Assess/discuss organization-level barriers and facilitators (8) 5. Set practice goals (17) |
| *Phase 2: Role Clarification and Team Training*   1. Clarify care team roles/tasks (9) 2. Map and revise workflows (9) 3. Teach communication & problem-solving skills (12) 4. Problem-solve implementation challenges (7) | *Phase 2: Mentoring and Action Planning*   1. Identify leader strengths (17) 2. Align DOCC implementation with practice priorities (18) 3. Reinforce leader attention to DOCC & support of its use (18) 4. Identify needed resources (e.g., space, time, technology) (7) 5. Create action plan to reach goals (17) 6. Problem-solve implementation challenges (7) |
| *Phase 3: Coaching and Consultation*   1. Teach/review DOCC content (14) 2. Review registry use and provide feedback (16) 3. Discuss case progress & treatment challenges (10, 15) 4. Provide treatment recommendations (15) 5. Adapt DOCC to meet practice needs (10) 6. Problem-solve implementation challenges (7) | *Phase 3: Leveraging*   1. Promote modeling and reinforcement of DOCC delivery (18) 2. Enhance community partner linkages (20) 3. Review and improve billing practices (19) 4. Adapt DOCC to meet practice needs (10) 5. Review/revise goals (11) 6. Problem-solve implementation challenges (7) |
| *Phase 4: Sustaining*   1. Lead structured team debriefings (11, 13) 2. Review/revise goals (11) 3. Create plan for sustainability and quality improvement (7, 11) 4. Problem-solve implementation challenges (7) | *Phase 4: Marketing and Sustaining*   1. Promote marketing/business care for DOCC (20) 2. Review lessons learned from other sites (20) 3. Create plan for sustainability and quality improvement (7, 11) 4. Update policies & procedures to enhance sustainability (17) 5. Review/revise goals (11) 6. Problem-solve implementation challenges (7) |

*Note: Numbers in parentheses indicate the implementation strategy each action is associated with (see Table 1)*
